# Knock-Out of Retrovirus Receptor Gene *Tva* in the Chicken Confers Resistance to Avian Leukosis Virus Subgroups A and K and Affects Cobalamin (Vitamin B_12_)-Dependent Level of Methylmalonic Acid

**DOI:** 10.3390/v13122504

**Published:** 2021-12-14

**Authors:** Anna Koslová, Pavel Trefil, Jitka Mucksová, Veronika Krchlíková, Jiří Plachý, Jakub Krijt, Markéta Reinišová, Dana Kučerová, Josef Geryk, Jiří Kalina, Filip Šenigl, Daniel Elleder, Viktor Kožich, Jiří Hejnar

**Affiliations:** 1 Institute of Molecular Genetics, Czech Academy of Sciences, Videnska 1083, 142 20 Prague, Czech Republic; anna.lounkova@gmail.com (A.K.); veronika.krchlikova@img.cas.cz (V.K.); jiri.plachy@img.cas.cz (J.P.); Marketa.Reinisova@img.cas.cz (M.R.); dana.kucerova@img.cas.cz (D.K.); geryk@img.cas.cz (J.G.); Filip.Senigl@img.cas.cz (F.Š.); daniel.elleder@img.cas.cz (D.E.); 2 BIOPHARM, Research Institute of Biopharmacy and Veterinary Drugs, Pohoří-Chotouň 90, 254 49 Jílové u Prahy, Czech Republic; pavel.trefil@bri.cz (P.T.); jitka.mucksova@bri.cz (J.M.); jiri.kalina@bri.cz (J.K.); 3 Department of Pediatrics and Inherited Metabolic Disorders, Charles University, First Faculty of Medicine and General University Hospital in Prague, 128 08 Prague, Czech Republic; jakub.krijt@lf1.cuni.cz (J.K.); Viktor.Kozich@vfn.cz (V.K.)

**Keywords:** avian leukosis virus subgroups A/K, *tva*, gene editing in chicken, vitamin B_12_/cobalamin

## Abstract

The chicken Tva cell surface protein, a member of the low-density lipoprotein receptor family, has been identified as an entry receptor for avian leukosis virus of classic subgroup A and newly emerging subgroup K. Because both viruses represent an important concern for the poultry industry, we introduced a frame-shifting deletion into the chicken *tva* locus with the aim of knocking-out Tva expression and creating a virus-resistant chicken line. The *tva* knock-out was prepared by CRISPR/Cas9 gene editing in chicken primordial germ cells and orthotopic transplantation of edited cells into the testes of sterilized recipient roosters. The resulting *tva* −/− chickens tested fully resistant to avian leukosis virus subgroups A and K, both in in vitro and in vivo assays, in contrast to their susceptible *tva* +/+ and *tva* +/− siblings. We also found a specific disorder of the cobalamin/vitamin B_12_ metabolism in the tva knock-out chickens, which is in accordance with the recently recognized physiological function of Tva as a receptor for cobalamin in complex with transcobalamin transporter. Last but not least, we bring a new example of the de novo resistance created by CRISPR/Cas9 editing of pathogen dependence genes in farm animals and, furthermore, a new example of gene editing in chicken.

## 1. Introduction

The retrovirus replication cycle starts with the attachment of a retrovirus particle to the host cell and reconformation of retroviral envelope (Env) glycoproteins. Reconformed Env molecules expose hydrophobic fusion peptides and insert them into the host cell cytoplasmic membrane. The process continues with fusion of both viral and host cell membranes and results in virus uptake, uncoating, reverse transcription, and trafficking toward the cell nucleus. Whatever details of the virus entry may differ between retrovirus genera, the Env reconformation is always primed by a lock–key interaction with a highly specific host cell receptor. Display of the proper receptor on the cell surface determines the host range and tissue specificity of a given retrovirus, and vice versa, the absence of receptor molecules means that a given cell or animal species is refractory to the virus infection.

Avian leukosis virus (ALV) exemplifies the principles of receptor-dependent virus entry. In total, seven ALV subgroups (A through E, J, and K) have been described in domestic chickens [1]. Each subgroup is highly specific as to the envelope glycoproteins and receptor usage. We now recognize four receptor proteins that are necessary for the host cell infection by ALV subgroups: Tva protein related to the low-density lipoprotein receptors (LDLR) [2,3], Tvb protein belonging to the tumor necrosis factor receptor family [4,5,6], Tvc protein similar to mammalian butyrophilins [7], and Tvj, which was identified as a Na^+^/H^+^ exchanger 1 [8]. For Tva, Tvb, and Tvc receptors, multiple mutated variants were identified that do not bind the ALV Envs because of frame-shift deletions or protein-fold changing amino acid substitutions, mostly cysteine or tryptophan [9,10,11]. Segregation of such resistant ALV receptor alleles has been observed, and susceptible/resistant breeds or inbred lines of chicken have been characterized.

ALV subgroup A and newly emerging subgroup K share the same Tva receptor [12]. Amino acid residues critical for ALV-A Env binding have been identified within the single cysteine-rich domain delineated between residues C11 and C50 [13,14,15]. No similar studies exist for ALV-K. In contrast to the *tva^s^* allele encoding, the standard Tva receptor sensitive to ALV-A/K, there are also receptor-defective alleles *tva^r^* (substitution C40W) and *tva^r^*^2^ (frame-shifting four-nucleotide insertion) that result in resistant birds when present in homozygous state in inbred chicken lines C and 7_2_, respectively [9]. In addition to the full resistance conferred by the *tva^r^* and *tva^r^*^2^ alleles, we described strongly decreased susceptibility to ALV-A in one semi-inbred chicken line and Indian red jungle fowl (*Gallus g. murghi*), which is the result of intronic deletions within the *tva* gene and inefficient splicing of the *tva* transcript [16]. Defective splicing due to the deletion of branch-point signal turned out to be also prevalent in outbred chickens [17]. Thus, natural polymorphisms within the *tva* gene could be used for breeding toward the resistance to ALV-A and ALV-K, two important concerns of the poultry industry. However, introducing an ALV-A-resistant allele into commercial chicken lines is a lengthy process, and a biotechnological shortcut such as CRISPR/Cas9-mediated knock-out or gene editing of *tva* gene might be much easier. Quite recently, such genetic knock-out has been demonstrated in cultured chicken cells by introduction of frame-shift mutations into the *tva* gene coding sequence [18,19].

The physiological function of Tva has remained at the level of speculation up to now. Its orthology with the mammalian CD320 (originally denoted 8D6A) gene [9] provided a clue when CD320 was identified as a cellular receptor for the uptake of cobalamin (cbl, vitamin B_12_)/transcobalamin II complex in humans [20]. This function of Tva has recently been confirmed by showing internalization of labeled cbl in chicken cells: the *tva* knock-out decreases cbl uptake ten times, whereas overexpression of tva displays a tenfold increase in cbl uptake [21]. Furthermore, ALV-A infection interferes with cbl uptake. Because cbl serves as an important metabolic regulator, and cbl deficiency manifests by apparent pathology in humans (reviewed in [22]), animal models are highly needed for comparative studies. A mouse model is already available [23] and demonstrates shifted values of intermediates in cbl-dependent metabolic cycles [24]. 

In the present study, we introduced a frame-shift mutation into the *tva* gene in chicken primordial germ cells (PGC) and obtained the in vivo genetic knock-out, *tva* KO, using the previously elaborated technique of orthotopic male germ line transplantation [25,26]. We employed a series of in vitro and in vivo techniques to show full resistance to infection with viruses of both ALV-A and ALV-K subgroup specificity. We detected increased levels of methylmalonic acid (MMA) in the sera of *tva* −/− chickens, which is consistent with MMA accumulation as a result of cbl depletion and inefficient conversion of methylmalonyl-CoA to succinyl-CoA. Thus, we demonstrated that the resistance to ALV-A and ALV-K could be engineered biotechnologically to establish an alternative model of mild cbl deficiency.

## 2. Materials and Methods

### 2.1. Ethical Statement

In this work, we conducted all experiments and procedures in accordance with the Czech legislation for animal handling and welfare. The Animal Commodities Department of the Ministry of Agriculture of the Czech Republic approved all animal experiments described in this study (approval no. 65823/2019-MZE-18134). Anesthesis of recipient roosters in the procedure of orthotopic transplantation was performed by intramuscular injection of 15 mg/kg ketamine (Narkamon, Bioveta, Czech Republic) and 4 mg/kg xylazine (Rometar, Bioveta, Czech Republic).

### 2.2. Experimental Chickens

Hybrid chicken embryos (♂ CC21 × L15 × ♀ outbred SH) were used as a source of PGCs, and hybrid recipient roosters (♂ CC21 × ♀ L15) were used for orthotopic PGC transplantation. SH hens were used for insemination. Inbred lines CC21, L15, and outbred population SH used for production of experimental chickens were maintained at the Institute of Molecular Genetics, Czech Academy of Sciences, Prague, Czech Republic [27]. Standard husbandry conditions applied in this work included 16 h light/8 h dark cycle, food/water provided ad libitum, and housing the birds in either deep litter individual cages (recipient roosters) or battery cages (inseminated hens). The chickens experimentally infected with replication-competent or tumor-inducing viruses were housed together and kept separate from the breeding birds. The laid eggs were incubated in a forced air incubator (BIOS MIDI).

### 2.3. PGC Cultivation and Tva Editing

**Figure 1:**
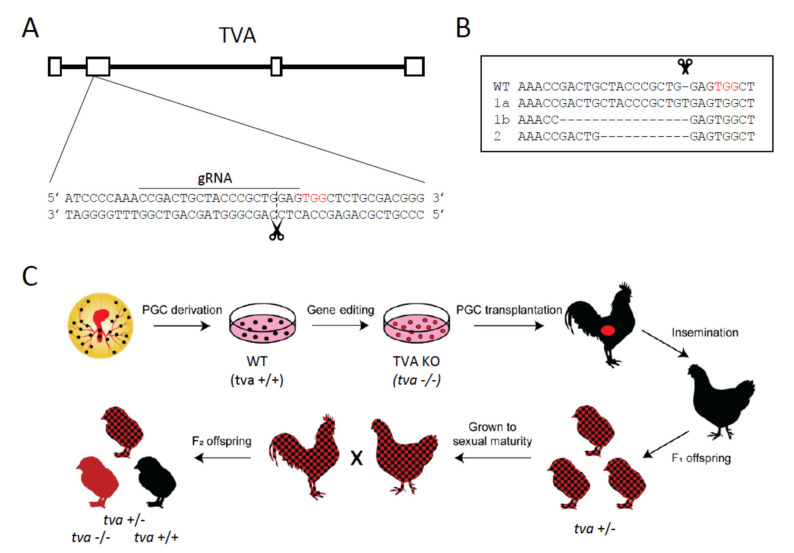
Generation of the *tva* gene knock-out in chicken PGCs and in vivo in the chicken. (**A**) Schematic representation of the *tva* coding sequence with exons and introns (top) and the design of gRNA used for CRISPR/Cas9 targeting of the *tva* gene (bottom). The target region of the *tva* gene is shown with the cleavage site (scissors), PAM sequence (in red), and gRNA sequence depicted with the line above. (**B**) Alignment of nucleotide sequences of the target region in wt and two PGC clones used for further work with out-of-frame deletions. (**C**) Schematic summary of the workflow and timeline during generation of *tva* −/− chickens.

We derived PGCs from the blood aspirations of WL/B chicken embryos incubated for 2.5 d, which corresponds to the Hamburger and Hamilton stage 16, using the procedures described in [28]. Briefly, we transferred 5 μL of blood from the dorsal aorta in a 48-well plate with 150 μL of Avian KO-DMEM (Thermo Fisher Scientific, Waltham, MA, USA) supplemented with growth factors, as described previously [29]. We expanded the PGCs for 2 to 3 weeks up to 10^5^ cells, isolated DNA from aliquots of PGCs using the PureGene kit (Qiagen, Hilden, Germany), and determined the sex by detection of the W chromosome. Oligonucleotide primers and cycling conditions used for W detection are described in [30]. One proliferative male PGC line was selected for the *tva* knock-out at the age of 52 d in culture. We used the PX458 vector pSpCas9BB-2A-GFP; AddGene no. 48138 [31] and prepared a CRISPR/Cas9 construct by cloning the *tva*-specific guide RNA sequence into the sg scaffold. The pX458-TVA construct [18] was designed to target the second exon of the *tva* gene (Figure 1A). PGCs (2.5 × 10^5^) were suspended in Nucleofector Solution V (Lonza) and mixed with 10 μg of pX458-TVA in total volume of 100 μL. Nucleofection was performed with the AMAXA nucleofector (Lonza) using the A-27 program. GFP-positive cells with the highest fluorescence intensity (top 5%) were single-cell-sorted 3 d post nucleofection and expanded for two weeks. The resulting clones were genotyped by amplification and sequencing the target region of the *tva* gene; oligonucleotide primers and cycling conditions used for *tva* characterization are described in [18].

### 2.4. Preparation of Sterile Recipient Roosters and PGC Transplantation

We used orthotopic PGC transplantation into roosters with suppressed endogenous spermatogenesis in order to obtain F1 offspring with the edited *tva* gene. The procedures of rooster irradiation and PGC transplantation were described previously [26,28,30]. Briefly, hybrids of inbred lines CC21I × L15 at the age of 8 months were irradiated with five doses of 8 Gy over two weeks using the Terabalt radiation unit (UJP Prague) with ^60^Co as a source of gamma rays. Subsequent decline of endogenous spermatogenesis was monitored in semen samples collected by dorsoabdominal massage. Azoospermic roosters were anesthetized and bilaterally injected through tunica albuginea with a dose of 1.9 × 10^6^ PGCs, clone 2 in 250 μL of culture medium. This transplanted PGCs clone spent 118 days in culture after the chicken embryo blood aspiration. We did not encounter any mortality or side effects associated with irradiation, anesthesia, or transplantation surgery.

### 2.5. Breeding of Tva KO Chickens

Appearance of PGC-derived spermatogenesis was monitored in semen samples collected by dorsoabdominal massage the third week after transplantation. The threshold for intramagnal insemination was the semen concentration of 10^4^ sperms per mL, and 0.1 to 0.4 mL of undiluted semen was used as the insemination dose. After successful intramagnal insemination, we also applied intravaginal insemination of SH line hens. The *tva* +/− genotype of F_1_ offspring was tested by PCR amplification as described above, grown for at least five months up to sexual maturity and mated in order to obtain F_2_ progeny segregating *tva* +/+, *tva* +/−, and *tva* −/− genotypes.

### 2.6. Off-Target Site Selection and Analysis

We used the CRISPOR tool (http://crispor.tefor.net/ (accessed on 12 September 2019)) for screening the NCBI chicken genome and selection of the most probable off-target sites. Candidate loci with the highest CFD scores, which contained at least 10 consecutive nucleotides matched with the 20-bp *tva* gRNA sequence and the NGG protospacer adjacent motif, were chosen for further analysis. PCR amplification primers were designed for each of the candidate off-target sites (Supplementary Figure S1), and the respective off-target regions were amplified using 100 ng of genomic DNA of the *tva* −/− PGC clone 2 and ejaculate of the recipient rooster No. 16. The PCR amplification was carried out under the following conditions: 3 min at 98 °C, 40 cycles of 10 s at 98 °C, 30 s at 62 °C, 30 s at 72 °C, and final amplification for 5 min at 72 °C. Sequence analysis of the resulting PCR products was performed, and the sequences were aligned to the respective NCBI *tva* sequence.

### 2.7. In Vivo Infection of Chickens and Viremia Analysis

*tva* +/+, *tva* +/−, and *tva* −/− chickens at the age of 23 to 39 days were infected by i.v. injection of 10^6^ IU of either RCASBP(A)GFP or RCASBP(K)GFP [12,16,32]. Nine and 13 d p.i., blood samples were collected, and sera were prepared. QIAamp Viral RNA Mini Kit (Qiagen) was used for virus RNA isolation from the chicken sera, according to the manufacturer’s protocol. Five microliters of prepared RNA was reversely transcribed with Protoscript II Reverse Transcriptase (NEB) and 3′CDS primer (5′AAGCAGTGGTATCAACGCAGAGTACT_30_VN3′). An amount of 1.5 μL of cDNA was then used for quantitative PCR with the MESA GREEN qPCR MasterMix Plus (Eurogentec) and primers for RCAS-A-env (Forward 5′GGGATGCGTAGGCTTCAGA3′, Reverse 5′AAAATCTGTAGCCATATGCACCG3′) or RCAS-K-env (Forward 5′GCCCCCGGAGCATTGACAA3´, Reverse 5′GGACCTGTCTGTGAACAATTATATAGC3′). To generate the standard curve for absolute quantification of gene expression, we used serial dilution of RCASBP-A and RCASBP-K plasmids. The samples were run in a Bio-Rad CFX96^TM^ Real-Time instrument with a 3-step protocol: one cycle of 8 min at 95 °C, then 40 cycles of 15 s at 95 °C, 25 s at 60 °C, and 35 s at 72 °C and final polymerization at 72 °C for 10 min. Cycles of quantification (Cq) values were generated by the CFX Manager software. The specificity of the PCR product was confirmed by melting curve analysis.

### 2.8. In Vitro Virus Spread in Infected Chicken Embryo Fibroblasts

Chicken embryo fibroblasts (CEFs) were individually derived from minced 10-day-old embryos, as described previously [32], and cultured in a mixture of two parts of DMEM and one part of F-12 medium supplemented with 5% calf serum (CS), 5% FCS, 1% chicken serum, and penicillin/streptomycin (100 μg/mL each) in a 5% CO2 atmosphere at 37 °C. The *tva* genotypes were determined by PCR and capillary sequencing. For PCR, we used primers TVA-fw (5′GCATGGTGCGGTTGTTGGAG3′) and TVA-rv (5′CTGTGCCGCCGGCGGTGGGC3′) with ExTaq HS DNA Polymerase (TaKaRa) and cycling conditions as follows: 98 °C for 3 min, 40 cycles of 98 °C for 10 s, 65 °C for 30 s, 72 °C for 30 s, and 72 °C for 5 min [16]. The RCASBP(A)GFP and RCASBP(K)GFP viruses transducing GFP were used for infection in 100 μL of media. Virus infection and in vitro spread of the virus were quantitatively assessed as an increasing percentage of GFP-positive cells by flow cytometry on day 3 p.i.

### 2.9. In Vivo Induction of Tumors with Transforming Virus Pseudotype

The transforming virus of A or K subgroup specificity was rescued from 16Q cells [33], which contain replication-defective and v-*src* containing BH-RSV. 16Q cells were co-cultured for 5 days with RCASBP(A)GFP- or RCASBP(K)GFP-infected DF-1 cells, and virus stocks consisting of GFP-reporter viruses and transforming viruses were harvested and frozen at −80 °C. The titer of the transformation component was assessed as 10^4^ and 10^5^ focus-forming units (FFU)/mL for subgroups A and K, respectively, by a focus assay in Brown Leghorn chicken embryo fibroblasts. *tva* +/+, *tva* +/−, and *tva* −/− chickens at the age of 23 to 39 days were inoculated with 500 or 1000 FFU of transforming virus in 0.1 mL of culture media subcutaneously into the left and right wing web, respectively. The sarcoma induction at the site of inoculation was followed and quantitated as the weight of tumors dissected from euthanized birds.

### 2.10. Quantification of MMA Serum Levels

MMA was determined by commercial kit ClinMass^®®^ Komplettkit, advanced (RECIPE Chemicals + Instruments, GmbH, München, Germany). LC-MS/MS analysis was carried out in Agilent 1290 Infinity LC System (Agilent Technologies, Palo Alto, CA, USA) coupled with an API 4000 triple quadrupole mass spectrometer with an electrospray ion source using the positive electrospray ionization technique and selected multiple reaction monitoring.

## 3. Results

### 3.1. Introduction of Tva Frame-Shift Deletions in Chicken PGCs

In order to generate *tva* −/− chicken PGCs, we used the *tva*-specific GFP-transducing CRISPR/Cas9 vector described in [18]. The CRISPR/Cas9 cleavage site in the second exon of *tva* and the guide RNA sequence are shown in Figure 1A. This vector was nucleofected into the PGCs derived from blood aspiration of 14- to 17-stage (H&H) donor male embryos. After nucleofection, we selected single-cell PGC clones and found two proliferative clones with indel mutations 5′ to the cleavage site. Clone No. 1 was heterozygous with insertion of one nucleotide (nt) in the first allele and deletion of 15 nt 5′ to the cleavage site in the second allele. Clone No. 2 was homozygous with both alleles bearing a frame-shifting deletion of 10 nt 5′ to the cleavage site in the second exon of *tva*. The extent of indels is shown in Figure 1B. The PGC clone No. 2 was expanded and used for further work. 

### 3.2. Generation of Tva KO, the Tva −/− Chicken Line

To generate knock-out chickens, we used the strategy of orthotopic transplantation of PGCs into the testes of adult recipient roosters and subsequent breeding of their offspring (Figure 1C). This original approach proved its efficiency in previous projects and provided transgenic and genetically edited chickens [26,30]. The PGC clone No. 2 was transplanted into two recipient roosters aged eight months. In order to replace the recipient’s own germ line with orthotopically transplanted manipulated PGCs, roosters were sterilized by gamma irradiation in advance, and the decline of endogenous spermatogenesis and the restoration of spermatogenesis after PGC transplantation were monitored. Both recipients restored spermatogenesis 10 to 11 weeks after transplantation. Following artificial insemination, both intramagnal and intravaginal, we obtained F_1_ offspring with the expected *tva* +/− genotype. We did not find any chicken of the wt *tva* +/+ genotype, which demonstrated the efficiency of the sterilization procedure and high replacement rate in grafted testes. The whole data on recipient cockerels and founder *tva* +/− heterozygotes are given in Table 1. After sexual maturation, F_1_ chickens were mated to produce F_2_ offspring, in which we observed segregation of *tva* +/+, *tva* +/−, and *tva* −/− genotypes (29:63:30, respectively) in correspondence with the expected Mendelian ratio (Pearson’s *X*^2^ test, *p* < 0.05).

### 3.3. Absence of Mutations at Potential Off-Target Sites

To assess the probability of non-specific non-*tva* mutations introduced into the genome by the CRISPR/Cas9 activity, we scanned the chicken genome using the CRISPOR tool and selected the most probable off-target sites. No potential off-target site with identical 20 nt core sequence was found; seven sites with the highest cutting frequency determination (CFD) score (Supplementary Table S1 and Figure S1) contained three to four mismatches. The nucleotide sequence of these potential off-target sites was analyzed in the DNA of the *tva* −/− PGC clone 2, which was used for generation of the *tva* −/− chicken line, and the DNA isolated from the ejaculate of recipient rooster No. 16. We did not identify any mutation in selected potential off-target sites (Supplementary Figure S2), which indicates that our *tva*-designed CRISPR/Cas9 construct hits specifically in the *tva* locus.

### 3.4. Tva −/− Chickens Are Resistant to Infection with ALV-A and ALV-K

**Figure 2:**
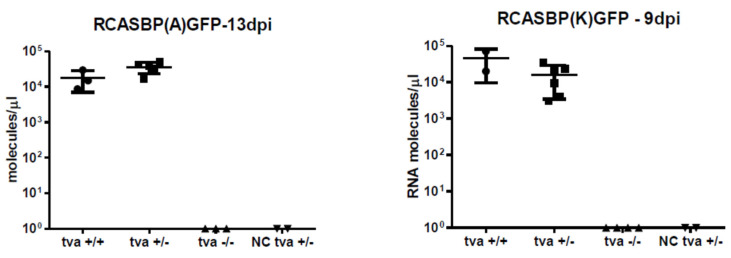
Resistance of *tva* KO chickens to in vivo infection with ALV subgroup A and K. *tva* +/+, *tva* +/−, and *tva* −/− chickens were infected with either RCASBP(A)GFP (**left**) or RCASBP(K)GFP (**right**) viruses. *tva* +/− chickens were also mock-injected as negative controls (NC). Primary viremia was analyzed by RT-PCR quantification of the respective *env* gene in the serum of RCASBP(A)GFP- or RCASBP(K)GFP-infected chickens. Each value represents the titer of viral RNA molecules as the mean of technical triplicates of individual chickens. For the groups of *tva* +/+ and *tva* +/− chickens, the means ± SD are shown.

Having the TvaKO F_2_ offspring segregating the wt *tva* +/+, heterozygotes *tva* +/−, and *tva* −/− knock-out chickens, we tested the resistance of *tva* −/− chickens to the infection with ALVs of A and K subgroup specificity. We challenged three to seven chickens of each three genotypes at the age of 23 to 29 days by intravenous (i.v.) injection of 10^6^ IU of either RCASBP(A)GFP or RCASBP(K)GFP vectors, which combine the backbone of replication- competent RCAS vector [32] with the *env* genes of respective subgroup specificity, and quantitatively tested by reverse transcriptase PCR (qRT PCR), whether primary viremia was established in the blood sera collected repeatedly during the experiment. On days 9 and 13 after infection with RCASBP(K)GFP or RCASBP(A)GFP, respectively, all *tva* +/+ and *tva* +/− chickens tested positive with 10^4^ to 10^5^ viral genomes per ml of sera, whereas *tva* −/− chickens and two mock-infected *tva* +/− chickens remained negative (Figure 2). In parallel, we tested the presence of replication-competent GFP-transducing virus in the blood sera of the same cohorts of chickens. Again, no infectious virus, either of A or K subgroup, was detected in *tva* −/− chickens, whereas all *tva* +/− and *tva* +/+ chickens displayed viremia, with the exception of one *tva* +/+ chicken. Titers 10^2^ to 10^4^ of the A subgroup virus were detected 7 and 14 days post infection. The K subgroup virus at titers 10^1^ to 10^3^ per ml was detected 7 days post infection and disappeared 14 days post infection in all chickens except one (Table 2).

### 3.5. In Vitro Cultured Tva −/− Embryo Fibroblasts Do Not Support the Spread of ALV-A and ALV-K

**Figure 3:**
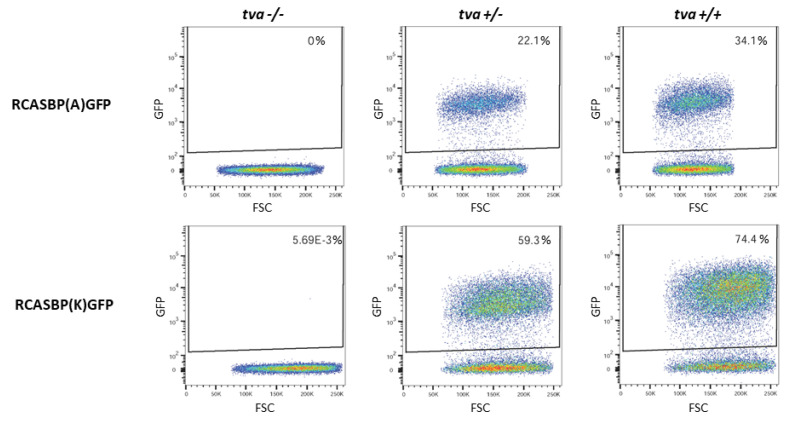
In vitro virus spread in embryo fibroblasts of tvaKO chickens. FACS dot-plots of *tva* −/− (left column), *tva* +/− (middle column), and *tva* +/+ chicken embryo fibroblasts infected with RCASBP(A)GFP (top row) or RCASBP(K)GFP reporter viruses. The percentages of gated GFP-positive cells are given in individual dot-plots. Representative examples of four *tva* −/−, two *tva* +/−, and two *tva* +/+ embryo fibroblast cultures are shown.

As an alternative assay demonstrating the resistance to ALV-A and ALV-K subgroups, we used the in vitro infection of chicken fibroblasts prepared individually from *tva* KO embryos of *tva* +/+, *tva* +/−, and *tva* −/− genotypes (two, two, and four embryos, respectively). We used GFP-transducing reporter viruses RCASBP(A)GFP and RACSBP(K)GFP for infection of cultured embryo fibroblasts, and the susceptibility to a given virus subgroup was estimated as the percentage of GFP-positive cells. Three days after infection with the RCASBP(A)GFP virus, we observed ca 35% of GFP-positive *tva* +/+ cells and 23% of GFP-positive *tva* +/− cells, whereas *tva* −/− cells remained GFP-negative (Figure 3). Similarly, infection with the RCASBP(K)GFP virus resulted in 65% to 70% of GFP-positive *tva* +/+ cells, 58% of GFP-positive *tva* +/− cells, and 0% in *tva* −/− cells. Although the multiplicity of infection was the same for both A and K subgroup viruses, we observed roughly two-fold more efficient virus spread in the case of RCASBP(K)GFP. This difference, however, was not observed in chicken DF-1 cells used as a positive control, where infection with RCASBP(A)GFP and RCASBP(K)GFP viruses resulted in roughly the same percentages of GFP-positive cells, 62.5% and 67%, respectively. The absence of virus spread in *tva* −/− chicken embryo fibroblasts confirms the resistance to ALV-A and ALV-K subgroups conferred by the knock-out of the chicken *tva* gene.

### 3.6. Tva KO Chickens Are Resistant to an ASLV-A/K-Pseudotyped Transforming Virus

**Figure 4:**
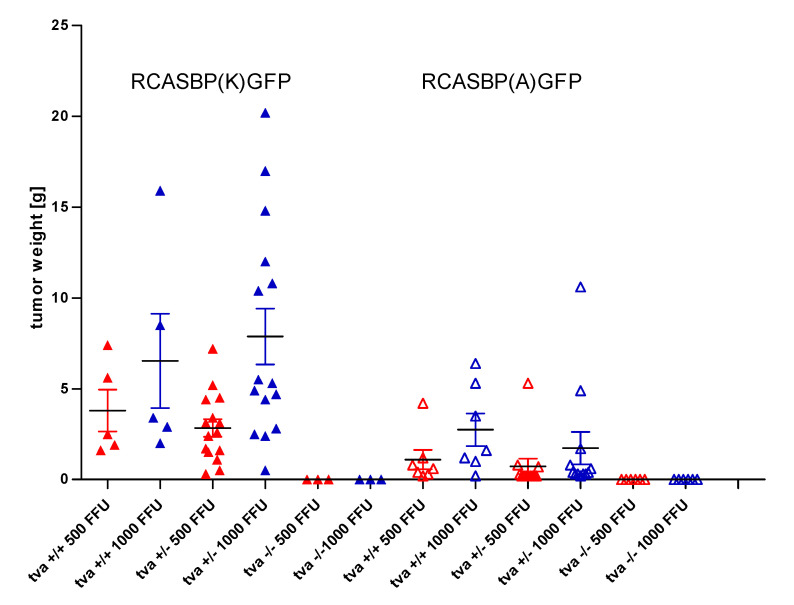
Resistance of *tva* KO chickens to in vivo tumor induction by transforming virus of K or A subgroup specificity. The v-*src* transducing virus pseudotyped with either K subgroup RCASBP(K)GFP (left, full triangles) or A subgroup RACSBP(A)GFP (right, open triangles) was applied in lower or higher dose of 500 FFU (red triangles) or 1000 FFU (blue triangles) to chickens of all genotypes (groups of 3 to 15 chickens). Weights of tumors dissected 16 d.p.i. are given as individual values and means ± SD.

Avian sarcoma and leukosis virus (ASLV) strains containing the v-*src* oncogene induce acute sarcomas in susceptible chickens, and the rapid growth of tumors depends on the receptor-mediated virus entry and spread. We therefore tested the virus resistance of *tva* −/− chickens by inducing tumors with a v-*src*-transducing virus pseudotyped with the A or K subgroup envelope. This assay had been established in our previous ALV receptor studies for subgroups A, B, C, D, E, and J [11,30]. The titer of the transforming pseudotyped virus was determined by an in vitro focus assay as focus-forming units (FFU), and each chicken in this experiment was inoculated with 500 FFU into one wing web and 1000 FFU into the other wing web. Five *tva* +/+, fifteen *tva* +/−, and three *tva* −/− chickens aged 23 to 39 days were inoculated with K subgroup-pseudotyped ASLV. All *tva* +/− and *tva* +/+ chickens developed rapidly growing tumors (Figure 4) 10 to 14 days after injection of both doses and had to be sacrificed 16 days p.i. In contrast, none of the three inoculated *tva* −/− chickens developed any tumor for the next four months and remained healthy, as confirmed by autopsy. Correspondingly, seven *tva* +/+, eleven *tva* +/−, and six *tva* −/− chickens aged 23 to 29 days were inoculated with A subgroup-pseudotyped ASLV, and all *tva* +/+ and *tva* +/− chickens developed tumors 10 to 16 days after injection of both higher and lower dose and had to be sacrificed 28 days p.i. Again, all *tva* −/− chickens remained healthy (Figure 4) and did not developed tumors for the next four months. The growth of tumors induced by A subgroup-pseudotyped ASLV was significantly slower than the growth of tumors induced by K subgroup-pseudotyped ASLV. In conclusion, these results confirm that the absence of Tva receptor confers resistance to ASLV subgroups A and K. 

### 3.7. TvaKO Chickens Have Increased Serum Levels of Methylmalonic Acid

**Figure 5:**
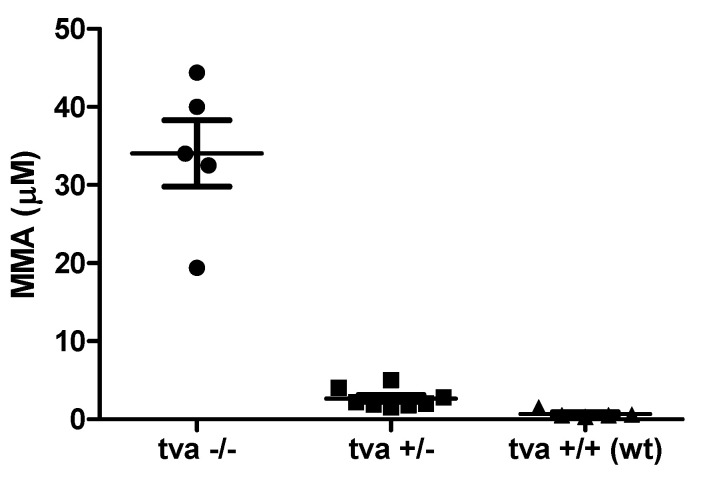
Serum levels of methylmalonic acid in *tva* −/− (circles), *tva* +/− (squares), and wild-type *tva* +/+ (triangles) chickens. Data are shown as serum levels (μmol per L) in individual chickens and means ± SD.

The physiological function of the Tva receptor was recently identified as the uptake of transcobalamin II bound cbl. In order to demonstrate that the cbl is affected in *tva* −/− chickens, we measured the serum levels of MMA, which is the most sensitive indicator of functional cbl depletion. In five *tva* −/− chickens, the levels of MMA reached 19 to 44 μmol/L, which is significantly higher than 1 to 5 μmol/L in eight *tva* +/− chickens and 1 to 2 μmol/L in five *tva* +/+ chickens (Figure 5). This finding is in accordance with our expectations, because methylmalonyl-CoA is the substrate for adenosylcobalamin-dependent conversion to succinyl-CoA, and MMA elevation in *tva* −/− chickens is congruent with its release from the accumulating reaction substrate under conditions of inefficient cbl uptake. 

## 4. Discussion

In this study, we demonstrated that homozygous in vivo knock-out of the chicken *tva* gene by a frame-shifting deletion in the second exon results in complete resistance to two ALV subgroups, A and K, which share Tva as an entry receptor. After ALV-J, where the complete resistance was achieved by gene editing of the chicken Na^+^/H^+^ exchanger 1 [30,34], it is the second example of artificial anti-viral resistance conferred by genetic manipulation of the chicken. In pigs, this strategy was employed for resistance to the porcine reproductive and respiratory syndrome virus and transmissible gastroenteritis virus by editing specific receptors CD136 [35] and pAPN [36]. The genetic manipulation of virus receptors is a promising anti-viral approach that targets the virus before it penetrates the host cell. Of course, before any practical application, other studies using field strains of the virus and commercial lines of chicken will be required. Another concern is the possibility that ALV-A could adapt to the *tva* deletion and overcome the resistance of TvaKO line. This possibility should be tested both in vitro and in vivo before the practical application of this strategy. 

In the chicken, however, this approach is hampered by the laboriousness and low efficacy of the procedures leading to the genetic knock-out or gene editing. This technology mostly relies on in vitro manipulation of PGCs, which must be reintroduced into embryos and matured to functional sperm [37]. Up to now, only a few examples of genetic knock-out have been reported [38,39]. Orthotopic transplantation of PGCs into the testes of sterilized recipients [26] has proven to be an efficient procedure in production of gene-edited poultry [30] and, at present, in the generation of the *tva* gene knock-out. Quite recently, another approach of CRISPR/Cas9 delivery into the chicken embryo blastoderm by adenoviral vector led to genetic knock-out of the melanophilin gene in quail and to the change in the plumage color [40]. Very promising and versatile resource is the Cas9 stably expressed in all tissues of transgenic animals, which facilitates in vivo organ-specific gene editing. This strategy was recently established in parallel in pigs and chickens [41]. The last addition to the PGC technology in the chicken is the system of inducible germ line ablation and introduction of donor genome-edited PGC into the surrogate host chicken [42]. 

In addition to its function as ALV receptor, Tva was recently identified as a specific receptor for cbl-saturated transcobalamin necessary for efficient cbl internalization into cells [21]. Thus, Tva ensures the bioavailability of cbl for internal conversions of MMA-CoA to succinyl-CoA and remethylation of homocysteine. Creation of the chicken *tva* knock-out is therefore interesting not only for the virus-resistant phenotype, but also as a potential model for the cbl metabolism. Genetic knock-out of orthologous CD320 in the mouse already exists [23], but the peculiarities of avian cbl uptake and transport, such as the absence of haptocorin (also known as transcobalamin 1) [43], and the importance of domestic poultry for human nutrition substantiate the use of a comparative chicken model. Genomic searches have shown that birds have only two cbl carrier proteins: transcobalamin (equivalent of mammalian transcobalamin 2) and gastric intrinsic factor [43,44], which binds cbl in the gastrointestinal tract and transports it to the blood. Since their separation from other vertebrate lineages, birds have lost haptocorrin, which in mammals is found in plasma, where it serves as a high affinity storage protein for cbl and its derivatives. Not unexpectedly, avian transcobalamin was found to display some protein motifs resembling haptocorrin and might partially substitute its role [43].

Another important cbl-dependent metabolic pathway is the conversion of homocysteine to methionine, where methyl-cbl serves as a methionine synthase cofactor necessary for the transfer of methyl groups from methyl tetrahydrofolate to homocysteine. Further conversion of methionine to S-adenosylmethionine, a universal donor of the methyl group [45], produces a critical substrate for biological methylation of proteins and nucleic acids. cbl depletion observed in the brains of CD320 KO mice was accompanied by strongly decreased global DNA methylation [46]. This suggests that blocks in the cbl uptake could affect epigenetic regulations throughout the genome and exert unexpected phenotypic changes in Tva- or CD320-depleted animals. For example, advanced cbl deficiency has been shown to contribute to nervous system demyelination and epigenetic factors such as histone modifications contribute to this phenotype [47].

The previously developed animal model of cbl depletion, the CD320 KO mouse, displayed specific neuropathology and behavioral alterations in addition to the aforementioned metabolic changes. Axonal demyelination in the spinal cord leading to partial loss of peripheral sensation [24] and the decreased brain mass and hippocampal nuclei were accompanied by anxiety and defects in learning, memory, and spatial orientation [48]. The exact phenomic analysis remains to be done in our comparative model. We did not observe any gross phenotypic effect segregating with the *tva* deficiency; the TvaKO chickens are normally fertile and do not display any special pathology. However, our analysis is based on a small number of outbred chickens available in the early phase of TvaKO line breeding, and slight effects on highly polymorphic traits such as growth or immunity cannot be analyzed in our system. Therefore, we will prepare an additional TvaKO line on the inbred background. Actually, the absence of specific growth retardation is true for already existing inbred chicken line 7_2_, bearing the frame-shifting four-nucleotide insertion in the *tva* gene [9], which has long been maintained in captivity without any deleterious phenotype. We, however, cannot exclude compensatory genetic changes in this case. The absence of distinct phenotype is consistent with the mouse model, where CD320 knock-out does not completely abrogate the cbl import, and clinically asymptomatic human cases with CD320 defect [49,50], implying a parallel transport mechanism [23,24]. An alternative cbl uptake pathway is also visible in the *tva* chicken cells, where the uptake of cbl-transcobalamin remains significantly above the background level [21]. MMA is the most sensitive marker of altered cbl metabolism, whereas other metabolites will be analyzed in the inbred Tva KO line. 

In vivo application of CRISPR/Cas9 in the livestock raises concerns of potential off-target mutations. Therefore, we selected the most probable off-target sites and analyzed them by PCR amplification and sequencing. We concluded that our CRISPR/Cas9 targeting of the chicken *tva* locus was very specific with no off-target mutations introduced into the loci with the highest CFD score. The ultimate solution would be to perform whole-genome analysis, applying next-generation sequencing technology to the freshly expanded PGC clones. On the other hand, we can expect that potential off-target mutations would disappear by random drift during the subsequent breeding of F_1_ and F_2_ offspring.

In conclusion, we suggest that the introduction of genome-edited chicken lines could help in reducing economic losses in the poultry industry, provided that Tva depletion does not affect the production performance. More precise modification of Tva altering a single amino acid residue critical for virus binding, e.g., C40W substitution present in the *tva^r^* allele [9], might be convenient for maintaining the normal production performance. On the other hand, the Tva knock-out generated by a frame-shifting deletion confirmed the cross-resistance to ALV-A and ALV-K and provided a new animal model for studies of cbl depletion in vivo. Although there exist naturally arisen virus-resistant *tva* alleles in inbred chicken lines, their introgression into commercial lines using conventional breeding would be a slow process that requires multiple rounds of backcrosses and could take many years. In the case of previously prepared resistance to ALV-J, we did not find any resistance-associated natural polymorphism in the chicken [51], and we identified the critical amino acid, W38, by comparison of resistant and susceptible galliform species [47,52,53]. The gene editing technology in combination with PGC orthotopic transplantation that we demonstrated in this and previous work [26,30] offers a one-generation shortcut of such lengthy process and allows for the maintenance of desirable qualities of the original line without gene penetration from the donor line. 

## Figures and Tables

**Table 1:** Summarized data on the PGC recipient roosters and founder *tva* +/− heterozygotes.

Rooster No. (No. of PGC Clone)	Restoration of Spermatogenesis (Weeks after Transplantation)	Hens Inseminated ^a^	Hatched Chickens (*tva* +/−) ^a^
1 (2) 16 (2)	10 11	2/3 4/3	8 (5♂/3♀) 6 (4♂/2♀)

^a^ Intramagnal/intravaginal insemination.

**Table 2:** Viremia in chickens inoculated with reporter ALV-A- and ALV-K-based vectors.

RCASBP(A)GFP			RCASBP(K)GFP		
Genotype	Chicken No.	Virus Titer ^a^	Genotype	Chicken No.	Virus Titer ^a^
*tva* −/−	52	0/0	*tva* −/−	129	0/0
*tva* −/−	58	0/0	*tva* −/−	150	0/0
*tva* −/−	62	0/0	*tva* −/−	155	0/0
*tva* +/−	51	10^2^/10^3^	*tva* +/−	125	10^3^/0
*tva* +/−	53	10^4^/10^3^	*tva* +/−	126	10^2^/0
*tva* +/−	55	10^3^/10^2^	*tva* +/−	127	10^1^/0
*tva* +/−	57	10^2^/10^3^	*tva* +/−	130	10^1^/0
*tva* +/−	59	10^3^/10^3^	*tva* +/−	151	10^2^/0
*tva* +/+	50	10^2^/10^3^	*tva* +/−	152	10^2^/10^1^
*tva* +/+	54	10^3^/10^4^	*tva* +/−	156	10^2^/0
*tva* +/+	61	0/10^2^	*tva* +/+	128	10^1^/0
			*tva* +/+	131	10^2^/0
			*tva* +/+	132	0/0
			*tva* +/+	133	10^1^/0
			*tva* +/+	134	10^1^/0
			*tva* +/+	148	10^2^/0
			*tva* +/+	154	10^2^/0

^a^ IU per mL 7 dpi/14 dpi.

## Data Availability

All data are contained within the article or Supplementary Materials.

## Supplementary Materials

The following are available online at https://www.mdpi.com/article/10.3390/v13122504/s1, Figure S1. Amplified nucleotide sequences of potential off-targets loci. Positions of primers used for PCR amplification (underlined) and sequences complementary to gRNA (bold) are shown. Figure S2. Nucleotide sequences of potential off-target sites amplified from PGC *tva* −/− clone 2 and ejaculate of recipient rooster No. 16 aligned to the NCBI chicken genome. Sequencing primers (either forward or reverse) are highlighted with green color and the gRNA complementary sequence with red color. Table S1. List of potential off-target sites with the highest CFD score and the core sequence of gRNA. The mismatches between gRNA and the off-target site are in bold, and the protospacer adjacent motifs are in italics.
