# Supplementary material for: Knock-Out of Retrovirus Receptor Gene Tva in the Chicken Confers Resistance to Avian Leukosis Virus Subgroups A and K and Affects Cobalamin (Vitamin B12)-Dependent Level of Methylmalonic Acid
Source: Viruses. 2021 Dec 14;13(12):2504. doi: 10.3390/v13122504 (PMC8708277; doi:10.3390/v13122504)
Supplement: Supplementary file 1 [file viruses-13-02504-s001.zip › viruses-1479637-supplementary.pdf]

Supplementary Figure S1. Amplified nucleotide sequences of potential off-targets loci. Positions of primers used for PCR amplification (underlined) and sequences complementary to gRNA (bold) are shown.

OFF-TARGET 1

mm4_intron_ESYT2_chr2_9588710
GAGCTGAAGTTCACCCTGCAGGGGAAGCCCGGCCTGCCGCCGCCTCCGCTGCTGTCGGGCCG
GCGGGCAGCGCCGGGCTCAGGCTAACGGCGACGGGGACGCGATGCCGGGGCGGAGGGATGCT
CCCCGGGCTCGCCCAACATCGCACCAGGCAGGACGTGCCCCCCGGGTACGGCATCCCCGCAC
CTCCTCCCTCCCGGCCCGCAG**CCAGCCGCTACCCGCAGGAGGGG**CCGAGGCCCCGGCCCGCC
CCACAGCCGCGCTGCCCGCCGGGCTCGGAGCCGCCTCGGAAGCGCCCGAAGGGTGTTGGCCG
CGCGCTCCCTCACTCACCCACGCAGGCAGATCGGCGGAGGAGACGCTGAGCCGCACCGCCTC
CTCCTCGTCCTCCAGGAAGGCGAGCGCCCGGCCCAGGCGGGAGGTCTTGCCGCCGCGGTGCC
GCCGGATCCAGAAGAGCCCGCACAGGGCGATGAGCACCCAGCTGAAGCTCAGCCCCAGGTA

1F GAGCTGAAGTTCACCCTGCA
1R TACCTGGGGCTGAGCTTCA

OFF-TARGET 2

mm4_intergenic_ENSGALT00000044901.2|VWA5B2_chr9_15903196
AAGCAGCGAACAGAACAGGAGCGGCAGAAAGTTCAGGTGCGGCTTCTAGGCTAGGGCTTTGC
CAGCAGGGTTTGCGTGGCCCAGCCAAAGCCGAGCGAGCGTCCGGGAGAGCGACGAGCGGCTC
TACTCCTCCTTGGCCCAGGCCGAGCCGGCGGCCCGCTGCTGCTGCAGGTGCGGAGCGACCGC
CTCGGTGTAGAACTTCTCCAGCTTGGGCACCGTCTTCCCCCAGAACTCGGCGTCGAACTCCA
CGGGCGCCACGGCCGTCTCCTTTTTGGTGTGCACCACGAAGTCGGCGTGCTGCAGGCCGGTG
GCCGCCAGCTGGCACTGCACCTGGGTGAAGTAGCCGTGATC**CCGCTTCAGCGCGTAGGAGTC**
GCCGTTCAGCTCCAGGCAGAAGTCCCTGTCCTTGCAGGCCTCGCGCACCGTCCTGTTGCGGT
GCTTGTAGGGACACTT

2F AAGCAGCGAACAGAACAGGA
2R AAGTGTCCCTACAAGCACCG

OFF-TARGET 3

mm4_intron_ENSGALT00000082879.1/ENSGALT00000074100.1_chr10_123
51360
AAGCCCCGAGGAACTAAGGAGAAAGTGAACGAAAGGCCGATTGCACGCAGCCACCTCCAAAG
CCACGAGCAAACCACGGCACCCAGCAGAGCCCAGGCGGGCGGTCAGCCTGCAGCT**CCCTCCC**
AGCGGGCAGCGGTCGGTGCCGGCAGCCGCACCCACCCCCCGGCAGGGGCAGCCCCAGCCCGG
CGGCACGGAGCTCCCATCGCCGCCTTTAGGGCAGCCCCCAGCCCAGCTGGGCTACAGACGAG
GAGCCCCGGGCCGGAGCGGCACCGCCTTCCTCTCCCTCTCTCCTCCTCCCCCCGCCAGCACT
GCCCACCCCCTCTCTCCCCGGAGGCCGTCACCGTATCGCCGTTGTCTGGGGACGCTCCTCGC
CGCCTCGGGCCGGGCTCCCAGCACCGCCAGCAGCACCAGCACCAGGGCGCAGCTCCTCGGGG
GGCCCGCGGCCGTCCCCAGCCGGGGGGGGCACCTGCAACACACACACG

3F AAGCCCCGAGGAACTAAGGA
3R CGTGTGTGTGTTGCAGGTG

OFF-TARGET 4

mm4_intergenic_HRH2|SPINK5_chr13_10537122

ACATTCCCAGCAGAAGAGGCAATTGGCTGATGCAGCGTTAGGCATTACTGCTCCCATCCCTG
AGGAGTGTGCTTTTGTGGTTCATCCCTTGCACCCAAATCACTCCTCAGTTACTCCACTCTGG
CCAATTGCTTTCACATCCTGCTGGCACTTCTAGGACTGTATTTTATGCTCTTGAATATAACA
TAAAGTCCT**CCCCTGCAGCGGGTAGCAATTTG**CAGCTGTATGCAAATTAAGTGCGTATTCCT
TTTCTCATGAATAATGGCCCTTGCAATGAGTTTTGAACTTTCTGCTCACTGCTCCCAAGGCA
AGAAGAGCTCAGAGTGCAGCAAGGGGTGGGGAGACGAGTGTGGCAGCCCCCAGACAACCAA
ATGTGGATGAACTGTTTGCAGGACCTGGATGGCCTCCGGGAGAGCCCTCCGCTGACCGTCCT
CCTCCGTGCATTTCA

4F ACATTCCCAGCAGAAGAGGC
4R TGAAATGCACGGAGGAGGAC

OFF-TARGET 5
mm4_intron_DGKK_chr4_1853057
TGTGCAGCTCCTTTACCTGGGAGTGAACTGTTGCTCTTTTGCTTCTGTGCCTTGTCCTTCCT
GGGCTTGGGGATGTTGAGGCGAAGCTTGAAGCTGCCCTTATTGGCACTGCTGGCACTCTCCT
GCAAGAAACAGGGATGAGCCCACCCCTACAGCCCCACAGCCCCCCCCCACCACCCCTATGCG
CCCACCTCCTCGCTGGGGTGGGCGATGGGTCCCAGCCAGTGGATGTCCAGCAGCCGCTGCAG
CTCCTGGCCCAACGCACTCAGGGGGCCCTGCAGCGCTTCCTCCTCCTGGGGTGAGTCCAACT
GCGGGGGACAGGGACCGTCCCCAGC**CCCACTGCCACCTGCTGGTGGGG**GGGACACCTGGGCC
GGAGGGCTCGGGGGGGTCCCCAGCCCCACACTCACCGCCTTCCCCTCCAGGAACGCTCTGGT
CTCGGTATAGAGCGCT

5F TGTGCAGCTCCTTTACCTGG
5R AGCGCTCTATACCGAGACCA

OFF-TARGET 6
mm4_exon_MIXL1_chr3_17105323
GTTCTCCTCCCACTCCGTTCCCAGCTCCGAGAA
GCCGCCGCCGGCCGGGAGGGGGAACGCGGGGCGGGGCGGGTAGGGCCCGGCCGGGGCGCCTC
CGCTCCCCCCGTGCGGGCGCAGCGCGGAGCGGGCGGGCCGGCGGCGCGGGGCGGCCACTCGC
GGTGCTCCTCCGGGCCGCGGAGCAGCGGGGCCGCGCCGCACGGCGAGCGCTGGGGCGGGGGC
GGTGGCGGTGCGGGGGCACCCGGCCGGGGCGGTCCCCGCTGCCGGCGAGACTTGGCGCGGCG
GTTCTGGAACCAGACCTGGGGAGAGAGGGCGGCCGTGAGGGCGCGGTGAGGGCGGGCGGCGA
GGAGGGCGGGCACCCCCTGCCCCTCACCTGGATGCGGGACTCGGGGATCTGCGTGGCGTCGG
CCAG**CCGCTCCCGCAGGTAGATGTCGG**GGTACATGGTGTCCTGGAACACCA

6F GTTCTCCTCCCACTCCGTTC
6R TGGTGTTCCAGGACACCATG

OFF-TARGET 7
mm4_intergenic_gga-mir-6560|ENSGALT00000022251.5_chr6_32490020
CTAAGCTGGGCGATAGTGCATGGCAAACAAAGCCTACACCATTTTATTTATGGCTGGCTATT
TACTTTATGCAACTCA**CCACTGCAGTGGGTATCAGTAGG**GATTTATAACAGATTGGATTTTC
GAAATGTTCATGTCCTCAGTGGCACACCATTGCTTTGTCAACTTTCACATCAGTTTTTCCCG
TGTCAGCTGGAACAGTCAGCAAACACCTAAAACTTGTAGTGTTGTTCCAGTTACAGGCATCT
GTGACTTGGAACCAGTCCAAAACATGCTGATCTACAGAACCACATCCACTGCCTTGCTGATA

CTTCCAGTTCTGCTTAGTGGTCCCAGTCATAGCAAGAGGCAACAAAAGTCAAAGCACAACGC
AAGGCCCTGCCGTGAGGAGTGAAGCATTCCAGCAGTGTAGCACAGTG

7F CTAAGCTGGGCGATAGTGCA

7R CACTGTGCTACACTGCTGGA

Supplementary Figure S2. Nucleotide sequences of potential off-target sites amplified from PGC *tva* -/- clone 2 and ejaculate of recipient rooster No. 16 aligned to the NCBI chicken genome. Sequencing primers (either forward or reverse) are highlighted with green color, the gRNA complementary sequence with red color.

```
OFF-TARGET 1
NCBI chicken        GAGCTGAAGTTCACCCTGCAGGGGAAGCCCGGCCTGCCGCCGCCTCCGCTGCTGTCGGGC
PGC                 ------------------------------------------------------------
ejaculate 16        --------------------GGGGAAGCCCGGCCTGCCGCCGCCTCCGCTGCTGTCGGGC


NCBI chicken        CGGCGGGCAGCGCCGGGCTCAGGCTAACGGCGACGGGGACGCGATGCCGGGGCGGAGGGA
PGC                 ------------------------------------------------------------
ejaculate 16        CGGCGGGCAGCGCCGGGCTCRGGCTAACGGCGACGGGGACGCGATGCCGGGGCGGAGGGA


NCBI chicken        TGCTCCCCGGGCTCGCCCAACATCGCACCAGGCAGGACGTGCCCCCCGGGTACGGCATCC
PGC                 ------------TCGCCCAACATCGCACCAGGCAGGACGTGCCCCCCGGGTACGGCATCC
ejaculate 16        TGCTCCCCGGGCTCGCCCAACATCGCACCAGGCAGGACGTGCCCCCCGGGTACGGCATCC
                                ************************************************

NCBI chicken        CCGCACCTCCTCCCTCCCGGCCCGCAGCCAGCCGCTACCCGCAGGAGGGGCCGAGGCCCC
PGC                 CCGCACCTCCTCCCTCCCGGCCCGCAGCCAGCCGCTACCCGCAGGAGGGGCCGAGGCCCC
ejaculate 16        CCGCACCTCCTCCCTCCCGGCCCGCAGCCAGCCGCTACCCGCAGGAGGGGCCGAGGCCCC
                    ************************************************************

NCBI chicken        GGCCCGCCCCACAGCCGCGCTGCCCGCCGGGCTCGGAGCCGCCTCGGAAGCGCCCGAAGG
PGC                 GGCCCGCCCCACAGCCGCGCTGCCCGCCGGGCTCGGAGCCGCCTCGGAAGCGCCCGAAGG
ejaculate 16        GGCCCGCCCCACAGCCGCGCTGCCCGCCGGGCTCGGAGCCGCCTCGGAAGCGCCCGAAGG
                    ************************************************************

NCBI chicken        GTGTTGGCCGCGCGCTCCCTCACTCACCCACGCAGGCAGATCGGCGGAGGAGACGCTGAG
PGC                 GTGTTGGCCGCGCGCTCCCTCACTCACCCACGCAGGCAGATCGGCGGAGGAGACGCTGAG
ejaculate 16        GTGTTGGCCGCGCGCTCCCTCACTCACCCACGCAGGCAGATCGGCGGAGGAGACGCTGAG
                    ************************************************************

NCBI chicken        CCGCACCGCCTCCTCCTCGTCCTCCAGGAAGGCGAGCGCCCGGCCCAGGCGGGAGGTCTT
PGC                 CCGCACCGCCTCCTCCTCGTCCTCCAGGAAGGCGAGCGCCCGGCCCAGGCGGTGAGCGTA
ejaculate 16        CCGCACCGCCT-------------------------------------------------
                    ***********

NCBI chicken        GCCGCCGCGGTGCCGCCGGATCCAGAAGAGCCCGCACAGGGCGATGAGCACCCAGCTGAA
PGC                 CGAACATCGTTAC-----------------------------------------------
ejaculate 16        ------------------------------------------------------------


NCBI chicken        GCTCAGCCCCAGGTA
PGC                 ---------------
ejaculate 16        ---------------


OFF-TARGET 2
NCBI chicken        AAGCAGCGAACAGAACAGGAGCGGCAGAAAGTTCAGGTGCGGCTTCTAGGCTAGGGCTTT
PGC clone 2         ------------------------------------GTGCGGCTTCTAGGCTAGGGCTTT
                                                        ************************

NCBI chicken        GCCAGCAGGGTTTGCGTGGCCCAGCCAAAGCCGAGCGAGCGTCCGGGAGAGCGACGAGCG
PGC clone 2         GCCAGCAGGGTTTGCGTGGCCCAGCCAAAGCCGAGCGAGCGTCCGGGAGAGCGACGAGCG
                    ************************************************************

NCBI chicken        GCTCTACTCCTCCTTGGCCCAGGCCGAGCCGGCGGCCCGCTGCTGCTGCAGGTGCGGAGC
PGC clone 2         GCTCTACTCCTCCTTGGCCCAGGCCGAGCCGGCGGCCCGCTGCTGCTGCAGGTGCGGAGC
                    ************************************************************
```

```
NCBI chicken          GACCGCCTCGGTGTAGAACTTCTCCAGCTTGGGCACCGTCTTCCCCCAGAACTCGGCGTC
PGC clone 2           GACCGCCTCGGTGTAGAACTTCTCCAGCTTGGGCACCGTCTTCCCCCAGAACTCGGCGTC
                      ************************************************************

NCBI chicken          GAACTCCACGGGCGCCACGGCCGTCTCCTTTTTGGTGTGCACCACGAAGTCGGCGTGCTG
PGC clone 2           GAACTCCACGGGCGCCACGGCCGTCTCCTTTTTGGTGTGCACCACGAAGTCGGCGTGCTG
                      ************************************************************

NCBI chicken          CAGGCCGGTGGCCGCCAGCTGGCACTGCACCTGGGTGAAGTAGCCGTGATCCCGCTTCAG
PGC clone 2           CAGGCCGGTGGCCGCCAGCTGGCACTGCACCTGGGTGAAGTAGCCGTGATCCCGCTTCAG
                      ************************************************************

NCBI chicken          CGCGTAGGAGTCGCCGTTCAGCTCCAGGCAGAAGTCCCTGTCCTTGCAGGCCTCGCGCAC
PGC clone 2           CGCGTAGGAGTCGCCGTTCAGCTCCAGGCAGAAGTCCCTGTCCTTGCAGGCCTCGCGCAC
                      ************************************************************

NCBI chicken          CGTCCTGTTGCGGTGCTTGTAGGGACACTT
PGC clone 2           CGTCCTGTTG--------------------
                      **********
```

OFF-TARGET 3

```
NCBI chicken          AAGCCCCGAGGAACTAAGGAGAAAGTGAACGAAAGGCCGATTGCACGCAGCCACCTCCAA
PGC clone 2           -------------------------------------CGATTGCACGCAGCCACCTCCAA
                                                           ***********************

NCBI chicken          AGCCACGAGCAAACCACGGCACCCAGCAGAGCCCAGGCGGGCGGTCAGCCTGCAGCTCCC
PGC clone 2           AGCCACGAGGAAACCACGGCACCCAGCAGAGCACAGGCGGGCGGTCAGCCTGCAGCTCCC
                      ********* ********************** ***************************

NCBI chicken          TCCCAGCGGGCAGCGGTCGGTGCCGGCAGCCGCACCCACCCCCCGGCAGGGGCAGCCCCA
PGC clone 2           TCCCAGCGGGCAGCGGTCGGTGCCGGCAGCCGCACCCACCCCCCGGCAGGGGCAGCCCCA
                      ************************************************************

NCBI chicken          GCCCGGCGGCACGGAGCTCCCATCGCCGCCTTTAGGGCAGCCCCCAGCCCAGCTGGGCTA
PGC clone 2           GCCCGGCGGCACGGAGCTCCCATCGCCGCCTTCAGGGCAGCCCCCAGCCCAGCTGGGCTA
                      ******************************** ***************************

NCBI chicken          CAGACGAGGAGCCCCGGGCCGGAGCGGCACCGCCTTCCTCTCCCTCTCTCCTCCTCCCCC
PGC clone 2           CAGACGAGGAGCCCCGGGCCGGAGCGGCACCGCCTTCCTCTCCCTCTCTCCTCCTCCCCC
                      ************************************************************

NCBI chicken          CGCCAGCACTGCCCACCCCCTCTCTCCCCGGAGGCCGTCACCGTATCGCCGTTGTCTGGG
PGC clone 2           CGCCAGCACTGCCCACCCCCTCTCTCCCCGGAGGCCGTCACCGTATCGCCGTTGTCTGGG
                      ************************************************************

NCBI chicken          GACGCTCCTCGCCGCCTCGGGCCGGGCTCCCAGCACCGCCAGCAGCACCAGCACCAGGGC
PGC clone 2           GACGCTCCTCGCCGCCTCGGGCCGGGCTCCCAGCACCGCCAGCAGCACCAGCACCAGGGC
                      ************************************************************

NCBI chicken          GCAGCTCCTCGGGGGGCCCGCGGCCGTCCCCAGCCGGGGGGGGGCACCTGCAACACACAC
PGC clone 2           GCAGCTCCTCGGGGGGCCCGCGGCCGTCCCCAGCCGGGGGGGGG----------------
                      ********************************************

NCBI chicken          ACG
PGC clone 2           ---
```

OFF-TARGET 4

```
NCBI chicken          ACATTCCCAGCAGAAGAGGCAATTGGCTGATGCAGCGTTAGGCATTACTGCTCCCATCCC
PGC                   -----------------------------------CGTTAGGCATTACTGCTCCCATCCC
PGC clone 2           -----------------------------------CGTTAGGCATTACTGCTCCCATCCC
                                                         *************************


NCBI chicken          TGAGGAGTGTGCTTTTGTGGTTCATCCCTTGCACCCAAATCACTCCTCAGTTACTCCACT
PGC                   TGAGGAGTGTGCTTTTGTGGTTCATCCCTTGCACCCAAATCACTCCTCAGTTACTCCACT
PGC clone 2           TGAGGAGTGTGCTTTTGTGGTTCATCCCTTGCACCCAAATCACTCCTCAGTTACTCCACT
```

```
                    ************************************************************

NCBI chicken        CTGGCCAATTGCTTTCACATCCTGCTGGCACTTCTAGGACTGTATTTTATGCTCTTGAAT
PGC                 CTGGCCAATTGCTTTCACATCCTGCTGGCACTTCTAGGACTGTATTTTATGCTCTTGAAT
PGC clone 2         CTGGCCAATTGCTTTCACATCCTGCTGGCACTTCTAGGACTGTATTTTATGCTCTTGAAT
                    ************************************************************

NCBI chicken        ATAACATAAAGTCCTCCCCTGCAGCGGGTAGCAATTTGCAGCTGTATGCAAATTAAGTGC
PGC                 ATAACATAAAGTCCTCCCCTGCAGCGGGTAGCAATTTGCAGCTGTATGCAAATTAAGTGC
PGC clone 2         ATAACATAAAGTCCTCCCCTGCAGCGGGTAGCAATTTGCAGCTGTATGCAAATTAAGTGC
                    ************************************************************

NCBI chicken        GTATTCCTTTTCTCATGAATAATGGCCCTTGCAATGAGTTTTGAACTTTCTGCTCACTGC
PGC                 GTATTCCTTTTCTCATGAATAATGGCCCTTGCAATGAGTTTTGAACTTTCTGCTCACTGC
PGC clone 2         GTATTCCTTTTCTCATGAATAATGGCCCTTGCAATGAGTTTTGAACTTTCTGCTCACTGC
                    ************************************************************

NCBI chicken        TCCCAAGGCAAGAAGAGCTCAGAGTGCAGCAAGGGGTGGGGGAGACGAGTGTGGCAGCCC
PGC                 TCCCAAGGCAAGAAGAGCTCAGAGTGCAGCAAGGGGTGGGGGAGACGAGTGTGGCAGCCC
PGC clone 2         TCCCAAGGCAAGAAGAGCTCAGAGTGCAGCAAGGGGTGGGGGAGACGAGTGTGGCAGCCC
                    ************************************************************

NCBI chicken        CCAGACAACCAAATGTGGATGAACTGTTTGCAGGACCTGGATGGCCTCCGGGAGAGCCCT
PGC                 CCAGACAACCAAATGTGGATGAACTGTTTGCAGGACCTGGATGGCCTCCGGGAGAGCCCT
PGC clone 2         CCAGACAACCAAATGTGGATGAACTGTTTGCAGGACCTGGATGGCCTCCGGGAGAGCCCT
                    ************************************************************

NCBI chicken        CCGCTGACCGTCCTCCTCCGTGCATTTCA
PGC                 CCGCTGACC--------------------
PGC clone 2         CCGCTGACC--------------------
                    *********
```

OFF-TARGET 5

```
PGC clone 2         ----------------------------------------GCTTCTGTGCCTTGTCCTTC
NCBI chicken        TGTGCAGCTCCTTTACCTGGGAGTGAACTGTTGCTCTTTTGCTTCTGTGCCTTGTCCTTC
                                                            ********************

PGC clone 2         CTGGGCTTGGGGATGTTGAGGCGAAGCTTGAAGCTGCCCTTATTGGCACTGCTGGCACTC
NCBI chicken        CTGGGCTTGGGGATGTTGAGGCGAAGCTTGAAGCTGCCCTTATTGGCACTGCTGGCACTC
                    ************************************************************

PGC clone 2         TCCTGCAAGAAACAGGGATGAGCCCACCCCTACAGCCCCACAGCCCCCCCCCACCACCCC
NCBI chicken        TCCTGCAAGAAACAGGGATGAGCCCACCCCTACAGCCCCACAGCCCCCCCCCACCACCCC
                    ************************************************************

PGC clone 2         TATGCGCCCACCTCCTCGCTGGGGTGGGCGATGGGTCCCAGCCAGTGGATGTCCAGCAGC
NCBI chicken        TATGCGCCCACCTCCTCGCTGGGGTGGGCGATGGGTCCCAGCCAGTGGATGTCCAGCAGC
                    ************************************************************

PGC clone 2         CGCTGCAGCTCCTGGCCCAATGCATTCAGGGGGCCCTGCAGCGCTTCCTCCTCCTGGGGT
NCBI chicken        CGCTGCAGCTCCTGGCCCAACGCACTCAGGGGGCCCTGCAGCGCTTCCTCCTCCTGGGGT
                    ******************** *** ***********************************

PGC clone 2         GAGTCCAACTGCGGGGGACAGGGACCGTCCCCAGCCCCACTGCCACCTGCTGGTGGGGGG
NCBI chicken        GAGTCCAACTGCGGGGGACAGGGACCGTCCCCAGCCCCACTGCCACCTGCTGGTGGGGGG
                    ************************************************************

PGC clone 2         GACACCTGGGCCGGAGGGCTCGGGGGGGTCCCCAGCCCCACACTCACCGCCTTCCCCTCC
NCBI chicken        GACACCTGGGCCGGAGGGCTCGGGGGGGTCCCCAGCCCCACACTCACCGCCTTCCCCTCC
                    ************************************************************

PGC clone 2         AGGAACGCTC--------------------
NCBI chicken        AGGAACGCTCTGGTCTCGGTATAGAGCGCT
                    **********
```

OFF-TARGET 7

```
PGC clone 2     --------------------TGGCAAACAAAGCCTACACCATTTTATTTATGGCTGGCTA
NCBI chicken    CTAAGCTGGGCGATAGTGCATGGCAAACAAAGCCTACACCATTTTATTTATGGCTGGCTA
PGC             --------------------TGGCAAACAAAGCCTACACCATTTTATTTATGGCTGGCTA
                                    ****************************************

PGC clone 2     TTTACTTTATGCAACTCACCACTGCAGTGGGTATCAGTAGGGATTTATAACAGATTGGAT
NCBI chicken    TTTACTTTATGCAACTCACCACTGCAGTGGGTATCAGTAGGGATTTATAACAGATTGGAT
PGC             TTTACTTTATGCAACTCACCACTGCAGTGGGTATCAGTAGGGATTTATAACAGATTGGAT
                ************************************************************

PGC clone 2     TTTCGAAATGTTCATGTCCTCAGTGGCACACCATTGCTTTGTCAACTTTCACATCAGTTT
NCBI chicken    TTTCGAAATGTTCATGTCCTCAGTGGCACACCATTGCTTTGTCAACTTTCACATCAGTTT
PGC             TTTCGAAATGTTCATGTCCTCAGTGGCACACCATTGCTTTGTCAACTTTCACATCAGTTT
                ************************************************************

PGC clone 2     TTCCCGTGTCAGCTAGAATAGTCAGCAAACACCTAAAACTTGTAGTGTTGTTCCAGTTAC
NCBI chicken    TTCCCGTGTCAGCTGGAACAGTCAGCAAACACCTAAAACTTGTAGTGTTGTTCCAGTTAC
PGC             TTCCCGTGTCAGCTGGAACAGTCAGCAAACACCTAAAACTTGTAGTGTTGTTCCAGTTAC
                ************** *** *****************************************

PGC clone 2     AGTCATCTGTGACTTGGAACCAGTCCAAAACATGCTGATCTACAGAACCACATCCACTGC
NCBI chicken    AGGCATCTGTGACTTGGAACCAGTCCAAAACATGCTGATCTACAGAACCACATCCACTGC
PGC             AGTCATCTGTGACTTGGAACCAGTCCAAAACATGCTGATCTACAGAACCACATCCACTGC
                ** *********************************************************

PGC clone 2     CTTGCTGGTACTGCCAGTTCTGCTTAGTGCTCCCAGTCATAGCAAGAGGCAACAAAAGTC
NCBI chicken    CTTGCTGATACTTCCAGTTCTGCTTAGTGGTCCCAGTCATAGCAAGAGGCAACAAAAGTC
PGC             CTTGCTGGTACTTCCAGTTCTGCTTAGTGGTCCCAGTCATAGCAAGAGGCAACAAAAGTC
                ******* **** ***************** ******************************

PGC clone 2     AAAGCACAACGCAAGG-------------------------------------------
NCBI chicken    AAAGCACAACGCAAGGCCCTGCCGTGAGGAGTGAAGCATTCCAGCAGTGTAGCACAGTG
PGC             AAAGCACAACGCAAGG-------------------------------------------
                *****
```

Supplementary Table S1. List of potential off-target sites with the highest CFD score and the core sequence of gRNA. The mismatches between gRNA and the off-target site are in bold and the protospacer adjacent motifs are in italics.

OFF-TARGET SITE NUMBER	SEQUENCE	CFD SCORE
tva	CCGACTGCTACCCGCTGGAG *TGG*	1.00
1	CC**AG**C**C**GCTACCCGC**A**GGAG ***G**GG*	0.38
2	**G**CGACT**C**CTAC**G**CGCTG**A**AG ***C**GG*	0.19
3	CCGAC**C**GCT**G**CCCGCTGG**GA** ***G**GG*	0.17
4	CC**C**ACTGC**C**ACC**T**GCTGG**T**G ***G**GG*	0.16
5	CCGAC**AT**CTACC**T**GC**G**GGAG ***C**GG*	0.13
6	CC**T**ACTG**A**TACCC**A**CTG**C**AG *TGG*	0.12
7	CC**T**ACTG**A**TACCC**A**CTG**C**AG *TGG*	0.11
